# The growth and evolution of cardiovascular magnetic resonance: a 20-year history of the Society for Cardiovascular Magnetic Resonance (SCMR) annual scientific sessions

**DOI:** 10.1186/s12968-018-0429-z

**Published:** 2018-01-31

**Authors:** Daniel C. Lee, Michael Markl, Erica Dall’Armellina, Yuchi Han, Sebastian Kozerke, Titus Kuehne, Sonia Nielles-Vallespin, Daniel Messroghli, Amit Patel, Tobias Schaeffter, Orlando Simonetti, Anne Marie Valente, Jonathan W. Weinsaft, Graham Wright, Stefan Zimmerman, Jeanette Schulz-Menger

**Affiliations:** 1 Division of Cardiology, Department of Medicine, Feinberg School of Medicine, Northwestern University, Chicago, IL USA; 2 Department of Radiology, Feinberg School of Medicine, Northwestern University, 737 N. Michigan Avenue Suite 1600, Chicago, IL 60611 USA; 3 Department of Biomedical Engineering, McCormick School of Engineering, Northwestern University, Evanston, IL USA; 4 Division of Biomedical Imaging, Leeds Institute of Cardiovascular and Metabolic Medicine, University of Leeds, Leeds, UK; 5 Cardiovascular Division, Department of Medicine, Perelman School of Medicine, University of Pennsylvania, Philadelphia, USA; 6 ETH Zurich, Zürich, Switzerland; 7 Charité – Medical University Berlin and German Heart Institute Berlin, Berlin, Germany; 8 National Heart, Lung and Blood Institute/ NIH, Bethesda, USA; 9 Charité – Medical University Berlin and German Heart Institute Berlin, Berlin, Germany; 10 University of Chicago, Chicago, IL USA; 11 Physikalisch-Technische Bundesanstalt, Berlin, Germany; 12 Kings College London, London, UK; 13 The Ohio State University, Columbus, OH USA; 14 Boston Children’s Hospital, Brigham & Women’s Hospital, Boston, USA; 15 Cornell University, Ithaca, NY USA; 16 Sunnybrook Research Institute, Toronto, Canada; 17 Johns Hopkins, Baltimore, MD USA; 18 Department of Cardiology and Nephrology, Charité – Universitätsmedizin Berlin, corporate member of Freie Universität Berlin, Humboldt-Universität zu Berlin, and HELIOS Klinikum Berlin Buch, Berlin, Germany

**Keywords:** SCMR, Archives, History, CMR, Trends, Cardiac, Heart

## Abstract

**Background and purpose:**

The purpose of this work is to summarize cardiovascular magnetic resonance (CMR) research trends and highlights presented at the annual Society for Cardiovascular Magnetic Resonance (SCMR) scientific sessions over the past 20 years.

**Methods:**

Scientific programs from all SCMR Annual Scientific Sessions from 1998 to 2017 were obtained. SCMR Headquarters also provided data for the number and the country of origin of attendees and the number of accepted abstracts according to type. Data analysis included text analysis (key word extraction) and visualization by ‘word clouds’ representing the most frequently used words in session titles for 5-year intervals. In addition, session titles were sorted into 17 major subject categories to further evaluate research and clinical CMR trends over time.

**Results:**

Analysis of SCMR annual scientific sessions locations, attendance, and number of accepted abstracts demonstrated substantial growth of CMR research and clinical applications. As an international field of study, significant growth of CMR was documented by a strong increase in SCMR scientific session attendance (> 500%, 270 to 1406 from 1998 to 2017, number of accepted abstracts (> 700%, 98 to 701 from 1998 to 2018) and number of international participants (42–415% increase for participants from Asia, Central and South America, Middle East and Africa in 2004–2017). ‘Word clouds’ based evaluation of research trends illustrated a shift from early focus on ‘MRI technique feasibility’ to new established techniques (e.g. late gadolinium enhancement) and their clinical applications and translation (key words ‘patient’, ‘disease’) and more recently novel techniques and quantitative CMR imaging (key words ‘mapping’, ‘T1’, ‘flow’, ‘function’). Nearly every topic category demonstrated an increase in the number of sessions over the 20-year period with ‘Clinical Practice’ leading all categories. Our analysis identified three growth areas ‘Congenital’, ‘Clinical Practice’, and ‘Structure/function/flow’.

**Conclusion:**

The analysis of the SCMR historical archives demonstrates a healthy and internationally active field of study which continues to undergo substantial growth and expansion into new and emerging CMR topics and clinical application areas.

## Background

On February 3, 2017, the Society for Cardiovascular Magnetic Resonance (SCMR) celebrated its 20th Annual Scientific Sessions in Washington D.C. Over the past 20 years, the field of cardiovascular magnetic resonance (CMR) has witnessed major advancements in data acquisition speed, image quality and development of novel imaging techniques [1–11], application of CMR to a broader range of cardiovascular diseases [12–25], and the incorporation into consensus statements [26–34] and clinical practice guidelines [26, 35–39].

On the direction of the SCMR Executive Committee, the SCMR Science Committee sought to evaluate research trends in CMR by evaluating session titles from SCMR Annual Scientific Sessions Programs over the past 20 years. Our goal was to track the number of abstracts and scientific contributions, analyze the evolution of research trends and hot topics, and identify the changes in main clinical focus areas and application areas over the past 20 years.

## Methods

### Data acquisition

Scientific programs from all SCMR Annual Scientific Sessions from 1998 to 2017 were obtained from the SCMR Headquarters Office and SCMR members. Digital program files were available for 2000–2017, while programs of the 1998 and 1999 annual scientific sessions were only available in paper form. SCMR Headquarters also provided data for number of attendees, country of origin of attendees (only available for 2004–2017) and number of accepted abstracts according to type (available for 1998–2018): oral, poster, walking poster, e-poster, moderated poster, and pre-conference workshop. In addition, the ratio of attendance / (number of accepted abstracts) was calculated.

### Data analysis

Session titles were abstracted from all programs and collated by year. Digital text analysis and visualization was performed using voyant-tools.org. The tool was used to visualize research and clinical trends by creating ‘word clouds’ representing the most frequently used key words in session titles in 5-year intervals. Common key words found in many session titles such as ‘MRI’, ‘CMR’, ‘cardiac’, and ‘MR’ were excluded from the analysis. To further evaluate research and clinical CMR trends over time, session titles were analyzed and subsequently manually sorted into 17 major subject categories. Title counts were grouped into four-year time periods: 1998–2001, 2002–2005, 2006–2009, 2010–2013, 2014–2017. The evolution of these major subject categories was sub-divided into “super growth” (an absolute increase of ≥ 8 sessions from the first time period to the last), and “strong growth” (an absolute increase of ≥ 4 sessions from the first time period to the last). In addition, new categories were defined as those that did not have any sessions in the first time period.

## Results

### SCMR annual scientific sessions 1998–2017: Location, attendance, abstracts

**Fig. 1:**
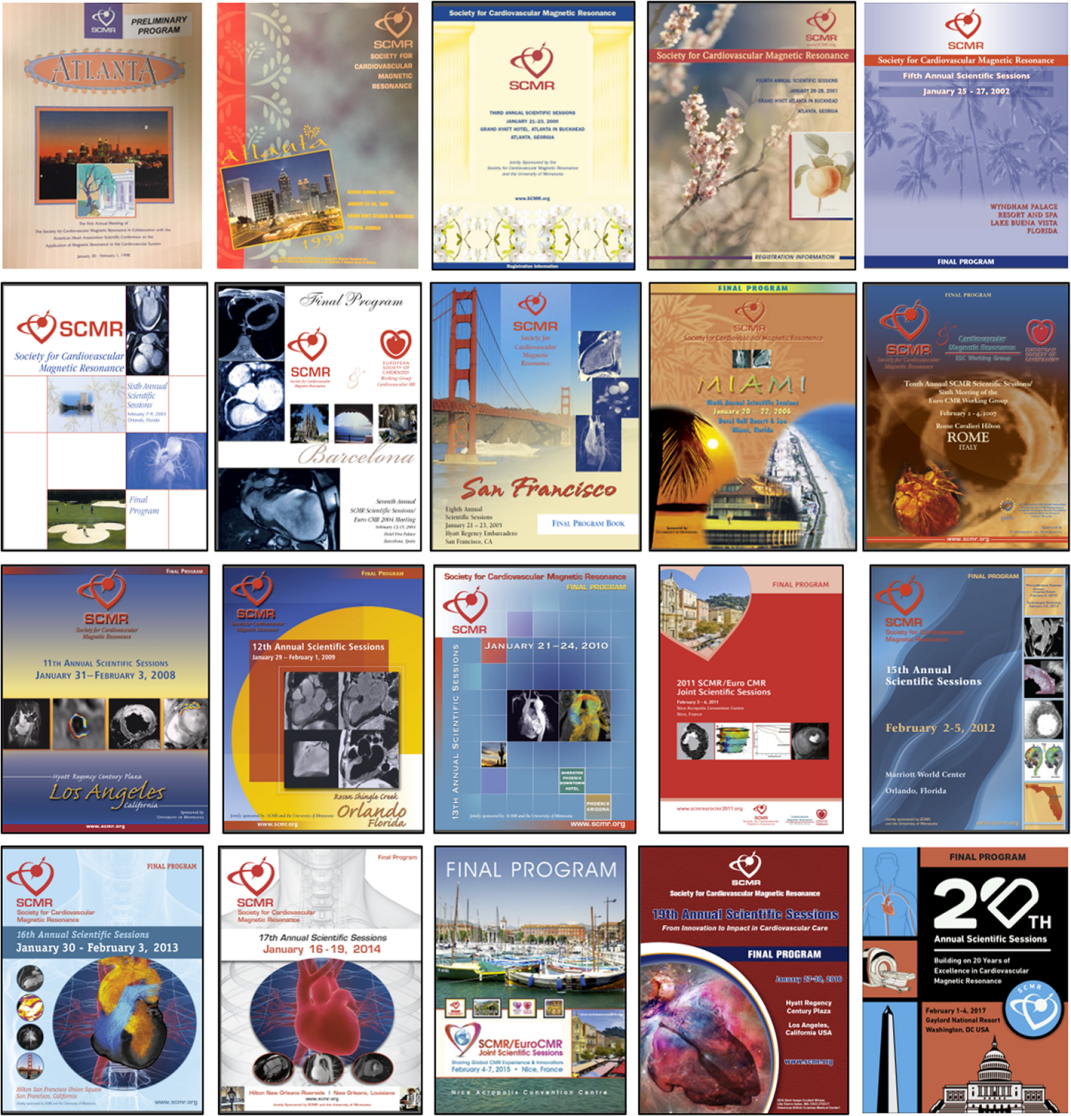
SCMR scientific sessions title pages from 1998 to 2017 illustrating a trend from basic to advanced CMR methods

**Fig. 2:**
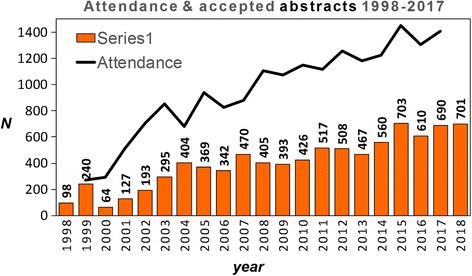
20-year evolution of SCMR annual scientific session attendance and number of accepted abstracts to be presented at the SCMR scientific sessions. Sustained growths of both annual scientific sessions attendance and scientific contributions are clearly evident. The attendance number of the 2018 meeting were not available at the time of this analysis

**Fig. 3:**
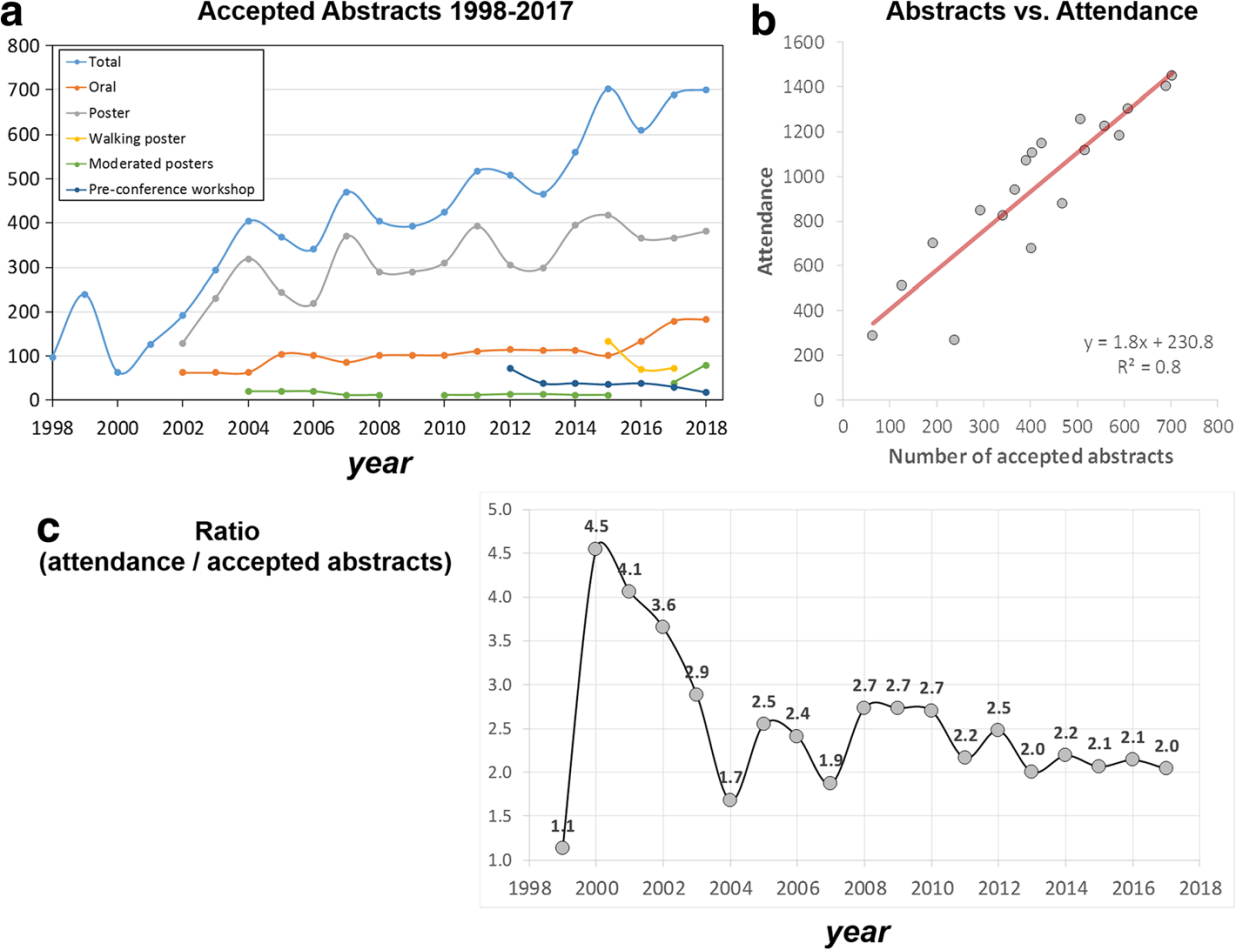
**a** Changes in number of accepted abstracts by type over the past 20 years. **b** Relationship between number of SCMR annual scientific session attendees and number of accepted abstracts. **c** Development of ratio of attendance vs. accepted abstract over the past 20 years

The dynamics and substantial growth of CMR research and applications are reflected in the evolution of SCMR annual scientific sessions locations and duration (Table 1), scientific session title pages from 1998 to 2017 (Fig. 1), and by the overall annual scientific sessions attendees, number of accepted abstracts over the past 20 years, and attendee / accepted abstracts ratio (Table 2 and Figs. 2 and 3). Figure 1 shows side-by-side comparisons of selected title pages of all 20 past SCMR annual scientific sessions. Style and illustrations reflect choices and preferences by the local organizers, scientific program committee, and SCMR board at the time of the annual scientific sessions. Nevertheless, tracking the temporal evolution of CMR images used for each title page provide an illustration of a trend from basic to advanced CMR methods.

**Table 1:** SCMR scientific session duration, annual scientific sessions location, and number of annual scientific sessions attendees 1998–2018

	SCMR annual scientific sessions			
Year	Duration [Days]	Location - City	Location - Country	Attendance
1998	3	Atlanta, Georgia	USA	N/A
1999	3	Atlanta, Georgia	USA	270
2000	3	Atlanta, Georgia	USA	291
2001	3	Atlanta, Georgia	USA	515
2002	3	Lake Buena Vista, Florida	USA	704
2003	3	Orlando, Florida	USA	850
2004	3	Barcelona	Spain	678
2005	3	San Francisco, California	USA	940
2006	3	Miami, Florida	USA	824
2007	3	Rome	Italy	879
2008	4	Los Angeles, California	USA	1107
2009	4	Orlando, Florida	USA	1074
2010	4	Phoenix, Arizona	USA	1150
2011	4	Nice	France	1117
2012	4	Orlando, Florida	USA	1258
2013	5	San Francisco, California	USA	1183
2014	4	New Orleans, Louisiana	USA	1226
2015	4	Nice	France	1451
2016	4	Los Angeles, California	USA	1305
2017	4	Washington DC	USA	1406
2018	4	Barcelona	Spain	N/A

*DC* District of Columbia, *USA* United States of America

**Table 2:** Number of attendees of SCMR scientific session by country of origin

	2004	2005	2006	2007	2008	2009	2010	2011	2012	2013	2014	2015	2016	2017	Average	Average I	Average II	Change [%]
	Barcelona	SF	Miami	Rome	LA	Orlando	Phoenix	Nice	Orlando	SF	New Orl.	Nice	LA	Wash DC	2004–2017	2004–2010	2011–2017	II vs. I
**Country**																		
United States	371	653	509	314	690	635	513	297	575	606	634	344	806	720	**547.6**	526.4	568.9	**8**
United Kingdom	72	49	49	100	86	83	84	170	114	164	124	299	105	143	**117.3**	74.7	159.9	**114**
Germany	82	81	50	129	103	67	58	122	59	77	58	151	74	64	**83.9**	81.4	86.4	**6**
Canada	9	24	34	38	82	69	52	45	75	93	77	50	75	90	**58.1**	44.0	72.1	**64**
Netherlands	57	29	15	71	50	55	36	102	38	40	44	108	30	45	**51.4**	44.7	58.1	**30**
Sweden	23	18	14	32	26	13	23	33	32	31	26	42	30	39	**27.3**	21.3	33.3	**56**
Switzerland	28	8	13	27	22	15	10	42	24	21	23	55	19	16	**23.1**	17.6	28.6	**63**
France	22	10	6	13	4	8	8	48	14	12	18	57	15	27	**18.7**	10.1	27.3	**169**
Japan	7	14	10	18	22	29	21	15	15	20	12	20	21	16	**17.1**	17.3	17.0	**−2**
Italy	14	9	8	47	10	12	7	38	7	9	0	31	15	12	**15.6**	15.3	16.0	**5**
Australia/NZ	14	5	4	13	12	5	10	24	15	21	8	28	12	11	**13.0**	9.0	17.0	**89**
Brazil/Colombia/Chile/Argentina	11	5	11	8	8	14	5	14	21	14	8	19	16	22	**12.6**	8.9	16.3	**84**
Spain	75	4	6	17	3	1	2	8	2	2	3	16	3	1	**10.2**	15.4	5.0	**− 68**
Belgium	17	7	5	20	4	4	3	15	1	4	2	18	1	3	**7.4**	8.6	6.3	**−27**
China/Hong Kong	4	5	0	1	6	3	2	11	2	11	1	10	18	13	**6.2**	3.0	9.4	**214**
South Korea	2	2	5	2	4	9	10	7	3	7	6	15	10	4	**6.1**	4.9	7.4	**53**
Austria	6	0	4	16	3	3	2	14	0	3	0	12	0	0	**4.5**	4.9	4.1	**−15**
UAE	3	0	0	2	5	0	0	15	2	0	4	24	3	2	**4.3**	1.4	7.1	**400**
Norway	12	3	1	7	6	1	2	4	4	2	2	8	3	2	**4.1**	4.6	3.6	**−22**
Greece	6	0	4	8	4	4	2	8	3	2	3	8	2	2	**4.0**	4.0	4.0	**0**
Portugal	6	0	0	15	2	0	0	14	1	1	2	14	0	0	**3.9**	3.3	4.6	**39**
Mexico	4	3	5	3	10	4	3	2	5	3	3	4	2	3	**3.9**	4.6	3.1	**−31**
Denmark	10	2	0	4	1	1	3	8	2	2	4	6	1	3	**3.4**	3.0	3.7	**24**
Poland	0	1	0	8	0	10	2	15	0	1	0	5	0	0	**3.0**	3.0	3.0	**0**
Singapore/Malaysia	3	1	1	5	0	1	1	5	1	1	0	10	0	1	**2.1**	1.7	2.6	**50**
Taiwan/Thailand	1	1	0	0	1	3	3	3	2	4	2	3	2	1	**1.9**	1.3	2.4	**89**
Israel	0	2	0	0	0	1	0	6	1	0	0	6	2	6	**1.7**	0.4	3.0	**600**
Hungary	5	0	0	4	2	0	0	4	0	0	0	3	0	2	**1.4**	1.6	1.3	**−18**
Finland	1	0	0	2	0	3	0	5	0	0	0	9	0	0	**1.4**	0.9	2.0	**133**
South Africa	0	0	1	0	0	0	0	1	0	2	2	4	2	7	**1.4**	0.1	2.6	**1700**
India	0	0	0	2	3	0	0	0	2	2	1	4	3	2	**1.4**	0.7	2.0	**180**
Ireland	1	0	0	1	0	2	0	2	4	0	0	3	0	2	**1.1**	0.6	1.6	**175**
Turkey	0	1	2	1	2	0	0		0	1	0	2	2	0	**0.8**	0.9	0.8	**−3**
Egypt	0	0	0	0	0	0	0	0	0	1	0	4	1	3	**0.6**	0.0	1.3	**100**
Czech Repulic	0	0	0	0	0	0	0	1	0	0	1	1	0	0	**0.2**	0.0	0.4	**40**
Russia	0	0	0	0	0	0	0	0	1	0	0	2	0	0	**0.2**	0.0	0.4	**40**
Iceland	0	0	0	0	0	0	1	0	0	0	0	1	0	0	**0.1**	0.1	0.1	**0**
Indonesia	0	0	0	0	0	0	0	0	0	0	0	0	0	1	**0.1**	0.0	0.1	**10**
Armenia	0	0	0	0	0	0	0	0	0	0	0	1	0	0	**0.1**	0.0	0.1	**10**
Bulgaria	0	0	0	0	0	0	0	0	0	0	0	1	0	0	**0.1**	0.0	0.1	**10**
**Region**																		
United States & Canada	380	677	543	352	772	704	565	342	650	699	711	394	881	810	**605.7**	570.4	641.0	**12**
Europe	437	221	175	521	326	282	243	652	306	371	309	850	298	361	**382.3**	315.0	449.6	**43**
Asia	17	23	16	28	36	45	37	41	25	45	22	62	54	38	**34.9**	28.9	41.0	**42**
Central & South America	15	8	16	11	18	18	8	16	26	17	11	23	18	25	**16.4**	13.4	19.4	**45**
Australia/NZ	14	5	4	13	12	5	10	24	15	21	8	28	12	11	**13.0**	9.0	17.0	**89**
Middle East & Africa	3	3	3	3	7	1	0	22	3	4	6	40	10	18	**8.8**	2.9	14.7	**415**

*LA* Los Angeles, *NZ* New Zealand, *SF* San Francisco, *UAE* United Arab Emirates

Significant growth of CMR research and clinical applications is corroborated by the consistent and strong increase of SCMR scientific session attendance and abstracts which document a > 500% growth in attendance (270 attendees in 1999 to 1406 in 2017) and a > 700% increase in the number of accepted abstracts (98 in 1998 to 701 in 2018). A more detailed breakdown of annual scientific sessions abstracts (oral and poster presentations) according to type is shown in Fig. 3 and illustrates that the growing number of contributions led to the creation of new categories (walking poster, e-poster, moderated poster) in recent years. As expected, a strong and significant relationship exists between the number SCMR annual scientific sessions attendees and abstract submissions (Fig. 3b). Interestingly, the attendance / accepted abstracts ratio varied considerably during early years (range from 1.1 to 4.5) but has stabilized over the past 7 years, ranging between 2.0 and 2.5.

Table 2 shows a detailed breakdown of SCMR annual scientific sessions attendees by country of origin over the past 14 years (2004–2017). There was a steady overall increase in international participation at the SCMR annual scientific sessions if average attendance in early (2004–2010) and more recent (2010–2017) time periods for the available data are compared; 570 to 641 (12% increase) for attendees from the United States and Canada, 315 to 450 for Europe (43% increase), 29 to 41 for Asia (42% increase), 13 to 19 for Central and South America (45% increase), 9 to 17 for Australia and New Zealand (89% increase), and 3 to 15 for Africa and Middle East (415% increase). As expected, SCMR annual scientific sessions held in Europe (2004, 2007, 2011, 2015) were characterized by an increase of European attendees compared to SCMR scientific sessions in the United States (see Table 2).

### Research and clinical trends: Key Word Extraction & Word Clouds

**Fig. 4:**
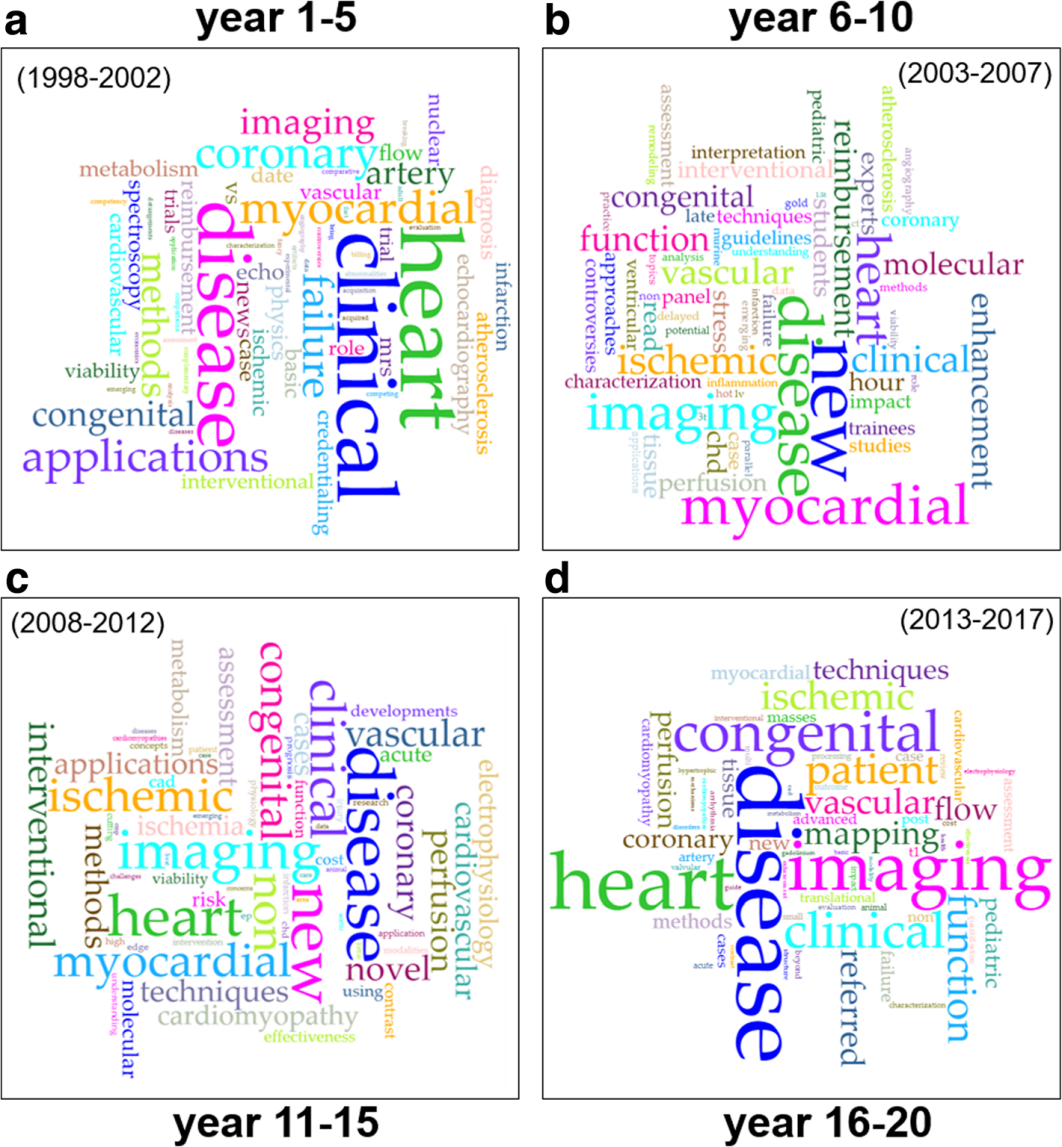
Word clouds visualizing the frequency of words used in SCMR scientific session titles over the past 20 years. Session titles were grouped in 5-year intervals and represent most frequently used words in SCMR session titles from 1998 to 2002 (**a**), 2003–2007 (**b**), 2008–2012 (**c**), and 2013–2017 (**d**). Word clouds were created using Voyant Tools (http://voyant-tools.org/)

Word clouds summarizing the results of key word extraction from SCMR annual scientific sessions titles are shown in Fig. 4. Results represent the most frequently used words in session titles (increase size indicated more frequent use) for four 5-year periods. Comparison of the temporal evolution of the word clouds over the past 20 years illustrates a topical shift from ‘CMR centric’ to ‘disease centric’. Initially, in years 1–5 (Fig. 4a) CMR techniques and their feasibility, the exploration of different application areas, and questions related to reimbursement were at the center of the SCMR annual scientific sessions. Subsequently, prominent key words such as ‘ischemic’ and ‘enhancement’ document the increasing importance of late gadolinium enhancement (Fig. 4b and c). In the past 10 years (Fig. 4c and d), the focus has shifted to clinical applications and translation (key words ‘patient’, ‘disease’, ‘congenital’, are more central). Finally, during the last 5 years (Fig. 4d), the corresponding word cloud reveals a renewed interest in novel techniques and quantitative CMR imaging techniques (more apparent role of key words ‘mapping’, ‘T1’, ‘flow’, ‘function’).

### CMR research trends and highlights at SCMR scientific sessions over the past 20 Years

**Fig. 5:**
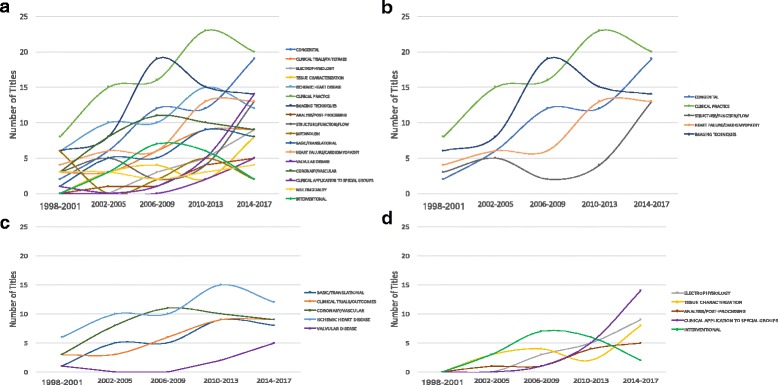
**a** Number of SCMR scientific session titles within major subject categories - trends over 20 years. **b** SCMR scientific session subject categories demonstrating “Super Growth” – defined as absolute growth ≥ 8 session titles over a 20-year time span. **c** SCMR scientific session subject categories demonstrating “Growth” – defined as absolute growth ≥ 4 session titles over a 20-year time span. **d** Several SCMR scientific session subject categories did not exist in the first time period, but exhibited significant growth in recent years

The number of sessions within all major subject categories over the past 20 years are depicted in Fig. 5a. Between the first time period (1998–2001) and the last (2014–2017), there was an increase in the number of sessions for every category except metabolism. The subject categories Clinical Practice, Congenital, Imaging Techniques, Heart Failure/Cardiomyopathy, and Structure/Function/Flow demonstrated “super growth” (Fig. 5b). The largest increase was seen in Congenital. Clinical practice had the most sessions in four of the five time periods. This category included sessions focusing on clinical cases, career development, global CMR, efficiency, safety, and cost. The category Structure/Function/Flow which has traditionally few sessions experienced “super growth” in the last time period due to sessions on strain, diastolic function, the right ventricle, and 4D flow. The subject categories Ischemic Heart Disease, Clinical Trials/Outcomes, Coronary/Vascular, and Basic/Translational demonstrated “strong growth” (Fig. 5c).

New categories (those that did not have any sessions in the first time period) include Electrophysiology, Tissue Characterization, Analysis/Post-processing, Interventional, and Clinical Application to Special Groups (Fig. 5d). This last category includes titles such as “Phenotyping and Risk Stratification in Hypertrophic Cardiomyopathy”, “Assessing the Hematology/Oncology Patient”, and “Cardiovascular Disease in Women – CMR’s Essential Role”.

Case sessions also saw significant growth in number and sophistication. From 1998 to 2001 there was a single case session offered, “Clinical Case Review Session: Bring Your Own”. From 2014 to 2017, there were 39 case sessions offered, including a live interventional CMR heart catheterization case presented by Children’s National Medical Center at the most recent SCMR annual scientific sessions in 2017. The first SCMR live case (real-time CMR-guided cardiac catheterization in an atrial septal defect patient) was a unique experience for the SCMR audience and a milestone in the history of the SCMR annual scientific sessions. SCMR attendees were provided with the opportunity to watch a clinical CMR catheterization program live in operation and recognize potential benefits of interventional CMR for pediatric and adult patients.

## Discussion

The results of our analysis of SCMR annual scientific sessions attendance, number and type of abstracts, as well as CMR research and application trends clearly demonstrated a healthy and internationally active field of study which continues to undergo substantial growth and expansion utilizing new and emerging CMR techniques to answer a broadening array of clinical questions. Changes in annual scientific sessions location and annual scientific sessions duration summarized in Table 1 are clear indications of CMR as a growing and international field of study. Initially varying attendees to accepted abstract ratio has stabilized in recent years above 2 indicating a strong and consistent interest in the SCMR annual scientific sessions also for clinicians and scientists who did not submit an abstract. These findings are supported by our key word extraction analysis which illustrated a shift from early focus on ‘MRI techniques and ‘feasibility’ to patient centered clinical translation and more recently novel techniques and quantitative CMR imaging. These changes were accompanied by a dramatic increase in the number of sessions from 46 in 1998–2001 to 168 in 2014–2017.

Nearly every topic category demonstrated an increase in the number of sessions from the first time period to the last. Clinical Practice led all categories in four of the five time periods. The top three growth areas were Congenital, Clinical Practice, and Structure/function/flow. In many sessions, growth mirrored the development of new imaging techniques or therapies. Tissue characterization has seen substantial growth since mapping techniques have become widely available. The advent of percutaneous valve techniques has coincided with the emergence of sessions dedicated to valve disease. Arrhythmias and CMR used to be mutually exclusive terms, but with real time techniques and recognition of the utility of CMR for defining the substrate for arrhythmias this area has blossomed. The “super growth” in the congenital category is not surprising, given the increasing role of CMR in this growing patient population. The category “Clinical Application to Special Groups” illustrates the growing application of CMR beyond traditional atherosclerotic coronary and vascular disease.

## Conclusion

We’ve seen the programs evolve from very broad sessions with a focus on development and validation, to a wide breadth of sessions that build upon the past and focus increasingly on specific applications to patient scenarios and groups where CMR might impact clinical care and practice guidelines. In this context, a continued collaboration between non-clinician PhD scientists and engineers and physician researchers and clinicians coupled with interactions with other clinically oriented societies will be critical for the continued success of CMR. The evolution of sessions at the SCMR annual scientific sessions mirrors the growth and maturation of the science and clinical practice of CMR over the past 20 years.

## Abbreviations

CMR: cardiovascular magnetic resonance
SCMR: Society for Cardiovascular Magnetic Resonance

## References

[1] Axel L, Otazo R (2016). Accelerated MRI for the assessment of cardiac function. Br J Radiol.

[2] Haaf P, Garg P, Messroghli DR, Broadbent DA, Greenwood JP, Plein S (2016). Cardiac T1 mapping and extracellular volume (ECV) in clinical practice: a comprehensive review. J Cardiovasc Magn Reson.

[3] Kim RJ, Shah DJ, Judd RM (2003). How we perform delayed enhancement imaging. J Cardiovasc Magn Reson.

[4] Kramer CM, Barkhausen J, Flamm SD, Kim RJ, Nagel E, Society for Cardiovascular Magnetic Resonance Board of Trustees Task Force on Standardized P (2008). Standardized cardiovascular magnetic resonance imaging (CMR) protocols, society for cardiovascular magnetic resonance: board of trustees task force on standardized protocols. J Cardiovasc Magn Reson.

[5] Kramer CM, Barkhausen J, Flamm SD, Kim RJ, Nagel E, Society for Cardiovascular Magnetic Resonance Board of Trustees Task Force on Standardized P (2013). Standardized cardiovascular magnetic resonance (CMR) protocols 2013 update. J Cardiovasc Magn Reson.

[6] Nayak KS, Nielsen JF, Bernstein MA, Markl M, Gatehouse P, Botnar R, Saloner D, Lorenz C, Wen H, Hu B, Epstein FH, J NO, Raman SV. Cardiovascular magnetic resonance phase contrast imaging. J Cardiovasc Magn Reson. 2015;17:71. 10.1186/s12968-015-0172-7 PMC4529988 26254979

[7] Oshinski JN, Delfino JG, Sharma P, Gharib AM, Pettigrew RI (2010). Cardiovascular magnetic resonance at 3.0 T: current state of the art. J Cardiovasc Magn Reson.

[8] Salerno M, Kramer CM (2013). Advances in parametric mapping with CMR imaging. JACC Cardiovasc Imaging.

[9] Tsao J, Kozerke S (2012). MRI temporal acceleration techniques. J Magn Reson Imaging.

[10] Tyler DJ, Hudsmith LE, Petersen SE, Francis JM, Weale P, Neubauer S, Clarke K, Robson MD (2006). Cardiac cine MR-imaging at 3T: FLASH vs SSFP. J Cardiovasc Magn Reson.

[11] Friedrich MG (2017). The future of cardiovascular magnetic resonance imaging. Eur Heart J.

[12] Bogaert J, Francone M (2009). Cardiovascular magnetic resonance in pericardial diseases. J Cardiovasc Magn Reson.

[13] Bradlow WM, Gibbs JS, Mohiaddin RH (2012). Cardiovascular magnetic resonance in pulmonary hypertension. J Cardiovasc Magn Reson.

[14] Butler CR, Thompson R, Haykowsky M, Toma M, Paterson I (2009). Cardiovascular magnetic resonance in the diagnosis of acute heart transplant rejection: a review. J Cardiovasc Magn Reson.

[15] Dormand H, Mohiaddin RH (2013). Cardiovascular magnetic resonance in Marfan syndrome. J Cardiovasc Magn Reson.

[16] Fieno DS, Saouaf R, Thomson LE, Abidov A, Friedman JD, Berman DS (2006). Cardiovascular magnetic resonance of primary tumors of the heart: a review. J Cardiovasc Magn Reson.

[17] Francis SA, Daly C, Heydari B, Abbasi S, Shah RV, Kwong RY (2013). Cost-effectiveness analysis for imaging techniques with a focus on cardiovascular magnetic resonance. J Cardiovasc Magn Reson.

[18] Maceira AM, Mohiaddin RH (2012). Cardiovascular magnetic resonance in systemic hypertension. J Cardiovasc Magn Reson.

[19] Maron MS (2012). Clinical utility of cardiovascular magnetic resonance in hypertrophic cardiomyopathy. J Cardiovasc Magn Reson.

[20] Myerson SG (2012). Heart valve disease: investigation by cardiovascular magnetic resonance. J Cardiovasc Magn Reson.

[21] Ntsinjana HN, Hughes ML, Taylor AM (2011). The role of cardiovascular magnetic resonance in pediatric congenital heart disease. J Cardiovasc Magn Reson.

[22] Parsai C, O'Hanlon R, Prasad SK, Mohiaddin RH (2012). Diagnostic and prognostic value of cardiovascular magnetic resonance in non-ischaemic cardiomyopathies. J Cardiovasc Magn Reson.

[23] Petitjean C, Rougon N, Cluzel P (2005). Assessment of myocardial function: a review of quantification methods and results using tagged MRI. J Cardiovasc Magn Reson.

[24] Raman SV, Aneja A, Jarjour WN (2012). CMR in inflammatory vasculitis. J Cardiovasc Magn Reson.

[25] Thornhill RE, Prato FS, Wisenberg G (2002). The assessment of myocardial viability: a review of current diagnostic imaging approaches. J Cardiovasc Magn Reson.

[26] American College of Cardiology Foundation Task Force on Expert Consensus D, Hundley WG, Bluemke DA, Finn JP, Flamm SD, Fogel MA, Friedrich MG, Ho VB, Jerosch-Herold M, Kramer CM, Manning WJ, Patel M, Pohost GM, Stillman AE, White RD, Woodard PK. ACCF/ACR/AHA/NASCI/SCMR 2010 expert consensus document on cardiovascular magnetic resonance: a report of the American College of Cardiology Foundation task force on expert consensus documents. J Am Coll Cardiol. 2010;55:2614-2662. 10.1016/j.jacc.2009.11.011 PMC3042771 20513610

[27] Dyverfeldt P, Bissell M, Barker AJ, Bolger AF, Carlhall CJ, Ebbers T, Francios CJ, Frydrychowicz A, Geiger J, Giese D, Hope MD, Kilner PJ, Kozerke S, Myerson S, Neubauer S, Wieben O, Markl M (2015). 4D flow cardiovascular magnetic resonance consensus statement. J Cardiovasc Magn Reson.

[28] Mavrogeni SI, Kitas GD, Dimitroulas T, Sfikakis PP, Seo P, Gabriel S, Patel AR, Gargani L, Bombardieri S, Matucci-Cerinic M, Lombardi M, Pepe A, Aletras AH, Kolovou G, Miszalski T, van Riel P, Semb A, Gonzalez-Gay MA, Dessein P, Karpouzas G, Puntmann V, Nagel E, Bratis K, Karabela G, Stavropoulos E, Katsifis G, Koutsogeorgopoulou L, van Rossum A, Rademakers F, Pohost G, Lima JA. Cardiovascular magnetic resonance in rheumatology: current status and recommendations for use. Int J Cardiol. 2016;217:135-148. 10.1016/j.ijcard.2016.04.158 27179903

[29] Messroghli DR, Moon JC, Ferreira VM, Grosse-Wortmann L, He T, Kellman P, Mascherbauer J, Nezafat R, Salerno M, Schelbert EB, Taylor AJ, Thompson R, Ugander M, van Heeswijk RB, Friedrich MG (2017). Clinical recommendations for cardiovascular magnetic resonance mapping of T1, T2, T2* and extracellular volume: a consensus statement by the Society for Cardiovascular Magnetic Resonance (SCMR) endorsed by the European Association for Cardiovascular Imaging (EACVI). J Cardiovasc Magn Reson.

[30] Moon JC, Messroghli DR, Kellman P, Piechnik SK, Robson MD, Ugander M, Gatehouse PD, Arai AE, Friedrich MG, Neubauer S, Schulz-Menger J, Schelbert EB, Society for Cardiovascular Magnetic Resonance I, Cardiovascular Magnetic Resonance Working Group of the European Society of C (2013). Myocardial T1 mapping and extracellular volume quantification: a Society for Cardiovascular Magnetic Resonance (SCMR) and CMR working Group of the European Society of cardiology consensus statement. J Cardiovasc Magn Reson.

[31] Nagel E, Lorenz C, Baer F, Hundley WG, Wilke N, Neubauer S, Sechtem U, van der Wall E, Pettigrew R, de Roos A, Fleck E, van Rossum A, Pennell DJ, Wickline S (2001). Stress cardiovascular magnetic resonance: consensus panel report. J Cardiovasc Magn Reson.

[32] Pennell DJ, Sechtem UP, Higgins CB, Manning WJ, Pohost GM, Rademakers FE, van Rossum AC, Shaw LJ, Yucel EK, European Society of c, Soceity for Cardiovascular Magnetic R (2004). Clinical indications for cardiovascular magnetic resonance (CMR): consensus panel report. J Cardiovasc Magn Reson.

[33] Schulz-Menger J, Bluemke DA, Bremerich J, Flamm SD, Fogel MA, Friedrich MG, Kim RJ, von Knobelsdorff-Brenkenhoff F, Kramer CM, Pennell DJ, Plein S, Nagel E (2013). Standardized image interpretation and post processing in cardiovascular magnetic resonance: Society for Cardiovascular Magnetic Resonance (SCMR) board of trustees task force on standardized post processing. J Cardiovasc Magn Reson.

[34] Friedrich MG, Kramer CM, Sodickson DK, Flamm SD, Buser P, Neubauer S, Scientific Program Committee of the Society for Cardiovascular Magnetic R (2007). Meeting highlights of the 10th annual scientific sessions of the Society for Cardiovascular Magnetic Resonance and 6th annual meeting of the working Group for Cardiovascular Magnetic Resonance of the European Society of Cardiology: Rome, Italy, February 2-4, 2007. J Am Coll Cardiol.

[35] Fratz S, Chung T, Greil GF, Samyn MM, Taylor AM, Valsangiacomo Buechel ER, Yoo SJ, Powell AJ (2013). Guidelines and protocols for cardiovascular magnetic resonance in children and adults with congenital heart disease: SCMR expert consensus group on congenital heart disease. J Cardiovasc Magn Reson.

[36] Hundley WG, Bluemke D, Bogaert JG, Friedrich MG, Higgins CB, Lawson MA, McConnell MV, Raman SV, van Rossum AC, Flamm S, Kramer CM, Nagel E, Neubauer S (2009). Society for Cardiovascular Magnetic Resonance guidelines for reporting cardiovascular magnetic resonance examinations. J Cardiovasc Magn Reson.

[37] Liu T, Pursnani A, Sharma UC, Vorasettakarnkij Y, Verdini D, Deeprasertkul P, Lee AM, Lumish H, Sidhu MS, Medina H, Danik S, Abbara S, Holmvang G, Hoffmann U, Ghoshhajra BB (2014). Effect of the 2010 task force criteria on reclassification of cardiovascular magnetic resonance criteria for arrhythmogenic right ventricular cardiomyopathy. J Cardiovasc Magn Reson.

[38] von Knobelsdorff-Brenkenhoff F, Pilz G, Schulz-Menger J (2017). Representation of cardiovascular magnetic resonance in the AHA / ACC guidelines. J Cardiovasc Magn Reson.

[39] von Knobelsdorff-Brenkenhoff F, Schulz-Menger J (2016). Role of cardiovascular magnetic resonance in the guidelines of the European Society of Cardiology. J Cardiovasc Magn Reson.

